# Colorectal cancer survivors’ long-term recollections of their illness and therapy up to seven years after enrolment into a randomised controlled clinical trial

**DOI:** 10.1186/s12885-023-10604-z

**Published:** 2023-02-13

**Authors:** Vinzenz Völkel, Brunhilde Steinger, Michael Koller, Monika Klinkhammer-Schalke, Patricia Lindberg-Scharf

**Affiliations:** 1 Tumour Centre Regensburg, Centre of Quality Management and Health Services Research, University of Regensburg, Am BioPark 9, 93053 Regensburg, Germany; 2 Centre for Clinical Studies, University Hospital Regensburg, Franz-Josef-Strauß-Allee 11, 93053 Regensburg, Regensburg, Germany

**Keywords:** Colorectal cancer, Quality of life, Long-term survival, Patient-reported outcomes, Patient-centred care

## Abstract

**Background:**

As a consequence of effective treatment procedures, the number of long-term survivors of colorectal cancer is ever increasing. Adopting the method of a previous study on breast cancer patients, the goal of the present research was to investigate colorectal cancer patients’ recollections of their illness and treatment experiences up to seven years after they have been enrolled in a randomised controlled clinical trial on the direct improvement of quality of life (RCT DIQOL).

**Methods:**

Colorectal cancer survivors in Bavaria, Germany were mailed a questionnaire on average 78·3 months after the start of their therapy and enrolment into RCT DIQOL. The questionnaire enquired about their worst experience during the colorectal cancer episode, positive aspects of the illness, and any advice they would give to newly diagnosed patients. Patient responses were categorised by two independent raters and cross-checked by a third independent rater. Frequencies of these categories were then quantitatively analysed using descriptive statistics.

**Results:**

Of 146 remaining survivors initially enrolled in RCT DIQOL, 96 (66%) returned the questionnaire. The majority (33%) of statements regarding the worst experience was referring to “psychological distress”, followed by “indigestion and discomfort during defecation” (17%), and “cancer diagnosis” (16%). Among survivors with history of a stoma, the majority (36%) regarded “stoma” as their worst experience. With 45%, “change in life priorities” has been the most frequent positive category before “support by physicians/ nurses” (25%). 43% of the survivors deemed “fighting spirit” as most important advice to overcome the disease.

**Conclusion:**

Even after many years, colorectal cancer survivors clearly remember experiences from the time of their illness. Echoing the results of the previous breast cancer survivors’ study, “psychological distress”, “change in life priorities” and “fighting spirit” emerged as prominent concepts. In addition, some aspects like the impact of a stoma are of specific importance for colorectal cancer survivors. These findings can be used to inform programmes to improve patient- and quality of life centred aftercare of tumour patients.

**Clinical trial information:**

NCT04930016, date of registration 18.06.2021.

## Background

In Germany, the lifetime risk of colorectal cancer ranges between 5.3% for women and 6.7% for men, corresponding to a nation-wide incidence of approximately 60.000 diagnoses per year. The relative survival rate [1] after five years for all stages is 65% among female and 63% among male patients [2]. Obviously, there is a huge group of “long-term survivors” which may be regarded as physically cured. However, various studies show that some cancer survivors suffer from permanent impairments of their quality of life (QoL) [3–6]. Many of them report that the illness inflicted a profound change on their life. They experienced their cancer diagnosis and the consecutive treatment as traumatic events [7, 8]: A systematic review of Jansen et al. reported that survivors had worse depression scores and suffer from long-term symptoms such as bowel problems and distress regarding cancer [9]. Lim et al. found out that bowel dysfunction causes functional limitations and negative QoL, while stomas pose a threat to the body image and confidence [10]. Generally, persisting physical symptoms hinder the return to work and contribute to increase financial burdens [10].

Among other important issues, a systematic review by Kotronulas et al. identified a substantial need of emotional support, information about diet/nutrition and long-term self-management of symptoms and complications [11]. It is the responsibility of health professionals not only to treat their patients’ cancer according to the latest guideline recommendations, but also to support them in preserving or improving their QoL. For this purpose, the Tumour Centre Regensburg developed a QoL pathway with systematic QoL diagnosis (the patients had to complete the European Organization for Research and Treatment of Cancer (EORTC) questionnaires QLQ-C30 (version 3.0) and QLQ-CR29 [12] 0–2 days before hospital discharge and 3, 6, 12, 18 months after surgery) and consecutive tailored QoL therapy (e.g., psychotherapy, physiotherapy, educational programmes for nutrition, etc.) for inpatient and outpatient care in a complex intervention [13, 14]. Two randomised trials (RCT DIQOL - randomised controlled clinical trial on the direct improvement of quality of life in colorectal and breast cancer patients using a tailored pathway with QoL diagnosis and therapy) in patients with colorectal [15] and breast cancer [16] demonstrated its effectiveness by showing a significantly better QoL in the intervention group patients.

This study is a questionnaire-based long-term follow-up of the colorectal cancer patient cohort that was originally enrolled in the RCT [15]. It aimed to learn more about long-term survivors’ subjective retrospective attitudes towards their illness and whether they can recall any particular negative or positive experiences associated with their cancer and its treatment. These recollections can give valuable insights into the long-term impact of a colorectal cancer disease. By communicating them to others, survivors inevitably influence future patients’ and physicians’ perspective towards the illness, which in turn will affect the acceptance and use of health- and supportive services. Findings of this sort can help physicians to understand their patients’ mindset better and inform efforts to improve communication patterns or provide additional supportive programmes during diagnosis, treatment, and aftercare of colorectal cancer.

## Methods

### Design

The aim of this cross-sectional, observational follow-up study of the RCT DIQOL [15] was to examine the recollections of colorectal cancer survivors more than five years after therapy onset regarding their (a) *worst experiences* during the illness, (b) potential *positive aspects* of the disease, and (c) the *advice they would give to future patients*. For this purpose, in April 2021, all survivors of the study sample were mailed by post a package of questionnaires supplemented by a stamped return envelope, a cover letter informing about the content and the aims of the study, a privacy policy, and an informed consent sheet. Survivors who did not respond within eight weeks received a mailed reminder.

### Sample

The study sample consisted of 220 primary colorectal cancer patients who had participated in the RCT DIQOL investigating the effectiveness of QoL diagnosis and therapy to improve patients’ QoL during the first 18 months after surgery [15]. All participants had been surgically treated between January 2014 and October 2015 in one of four participating certified colorectal cancer centres in Bavaria, Germany. To achieve high external validity, the trial inclusion criteria had no restrictions regarding disease stage or age. Details of this complex intervention can be found elsewhere [15, 17]. Follow-up of survivors of this sample was conducted between April 2021 and August 2021 about five to seven years after colorectal cancer diagnosis. Of the 220 patients enrolled into the randomised study, 62 had already died at the time of follow-up (survival information was provided by the clinical cancer registry Tumour Centre Regensburg, Centre of Quality Management and Health Services Research of the University of Regensburg, before conducting the follow-up survey) and twelve had refused further participation. Thus, 146 survivors were available for the follow-up study. Diverging from the definition provided by the National Cancer Institute [18], the term “survivor” in the context of this study is referring to a minimum survival period of five years after the cancer diagnosis. Applying this criterion makes it possible to concentrate on patients who seem to have overcome their disease permanently and is commonly accepted in cancer statistics [2, 19].

### Measures / instruments

#### Qualitative questionnaire

In addition to standardised questionnaires aiming to assess the actual QoL (EORTC QLQ-C30 and QLQ-CR29; not reported in this paper) the patient-reported outcome (PRO) form included a set of three qualitative, open-ended questions:

- *“Which has been the worst experience regarding your cancer disease?”*
- *“Have there also been positive aspects according to the illness?”*
- *“Which advice would you give newly diagnosed colorectal cancer patients to cope with the disease?”*

This questionnaire had been used beforehand in a survey with 133 breast cancer survivors of the same catchment area [20]. To be able to compare the statements of colorectal cancer survivors with those of breast cancer survivors, the same wording was used for these three questions in the present study.

#### Demographic and clinical data

Data were available from the earlier RCT DIQOL [15].

### Developing a category system for qualitative answers

To analyse the participants’ answers, a category system was developed using a three-step approach. To begin with, the applicability of the existing category system from the breast cancer survivors’ survey [20] was checked. Breast cancer specific categories like e.g. “mastectomy” were omitted. Subsequently, using a word-by-word analysis, the frequency of each single word in the survivors` answers was counted using a computer programme. Thus, issues which were commonly addressed by participants could be objectively identified. This procedure is based on “Linguistic Inquiry and Word Count” (LIWC) by Pennebaker et al. [21]. At last, two independent investigators conducted an inductive analysis encompassing all statements regarding the *worst experience*, *positive aspects*, and *advice for future patients* to create additional preliminary categories, where necessary. For this, the following criteria were considered:

- Each category should be broad enough to reflect a survivor’s perspective.
- At the same time categories were designed to be mutually exclusive and as specific as possible, to include meaningful information regarding the content of the data.

The definitive category system contains 13 different categories for the *worst experience*, nine categories for *positive aspects*, and 16 categories for *advice for future patients* (see Table 1; non pre-existing categories which have been created based on the word by word analysis and the inductive analysis are marked by an asterisk).

**Table 1:** Category system with (translated) sample statements

Category	Description	Sample statement (translated)
**Worst experience**		
Psychological distress	Anxiety or uncertainty about the course or outcome of the illness.	*“Fear that the tumour could already have spread, which was not the case though.”*
Indigestion and discomfort during defecation*	Discomfort during defecation (e.g. diarrhoea, incontinence) after primary surgery or stoma reversal and a necessary change in diet.	*“permanent diarrhoea”*
Cancer diagnosis	The shock of receiving cancer diagnosis and the fact of being a cancer patient.	*“the diagnosis itself!”*
Stoma*	The fact of having a stoma with related impairments.	*“carrying a stoma-pouch (detachment)”*
Hospital stay including surgery*	The time of hospital stay including surgical treatment.	*„cowardice of the doctors in the clinic, experience to be at someone’s mercy, helplessness”*
Chemotherapy	Chemotherapy with related side-effects.	*“chemotherapy”*
Financial burden*	Financial problems or worries caused by cancer diagnosis.	*„That I did not find a job afterwards, I am unemployed ever since.”*
Additional illnesses	Additional diseases like comorbidities or recurrence during or after colorectal cancer.	*„the fact that metastases recurred”*
Pain*	Any form of pain during the course of the illness.	*„unbearable pain“*
Nothing	No worst experience.	*“did not experience any discomfort”*
Social burden	Fear of family or other conflicts in partnership or family caused by the illness.	*“worrying for the family; have to carry my wife with her wheelchair to dialysis throughout the whole year”*
Radiotherapy	Radiotherapy with related side-effects.	*“radiotherapy”*
Other	Statements not fitting in one of the other categories	*“fatigue, grogginess”*
**Positive aspects**		
Change in life priorities	Change of one`s own priorities in life in terms of living life more consciously and relaxed, or changes in lifestyle.	*“One appreciates (healthy) everyday life again to a greater extent!”*
Support by physicians/ nurses	The good (medical) treatment by physicians or nurses.	*“outstanding care and advice provided by the doctors from the hospital* (name omitted)*”*
Social support	Support by family, friends, or colleagues as well as unexpected help from others.	*“I re-experienced the love of my wife.”*
Good course of cancer	The good course and outcome of the illness.	*“being completely cured”*
Gratitude	Being grateful to have survived.	*“I am really glad to have regained my physical health. Thanks to Prof.* (name omitted)*”*
Hope*	Being positive about the expected outcome	*“I was convinced to a 100% that I would overcome the illness.”*
Stoma reversal*	The reversal of the stoma.	*“having no stoma anymore“*
Rehabilitation*	To have been on rehabilitation	*“having been on rehab”*
Other	Statements not fitting in one of the other categories	*“I advise my acquaintances to participate in cancer screening programs.”*
**Advice**		
Fighting spirit	Think positive, fight, never lose hope, and be patient.	*“Giving up means loosing!“*
Cancer screening	Have regular cancer screening.	*“Please go to the screening colonoscopy!”*
Change in lifestyle*	Take the disease as an opportunity to adopt a healthier lifestyle.	*“Like I have already mentioned: I would advise a healthy diet and physical activity.”*
Confidence in physicians	Trust your physician and follow his/ her instructions.	*“Follow the doctors‘ advice!“*
Openness	Confide in somebody and talk a lot about the illness.	*“Talk to your family or friends about your fears!”*
Immediate treatment*	No hesitation in getting treatment right away.	*“early surgery”*
Information	Keep calm, get a second opinion, and inform yourself about the illness.	*“Look for different surgical options, consider different options; look for less invasive surgical techniques and prefer those!”*
Business as usual	Don`t think too much about the illness, live life in a normal way.	*“Continue living as usual!“*
Rehabilitation*	Go on rehabilitation.	*“Definitely go on rehab! This helped me a lot.”*
Acceptance	Accept the illness.	*“Accept the disease!“*
Support group	Visit a support group.	*“seeking information among fellow sufferers”*
No advice	It is not possible to give any advice for fellow patients.	*“no advice possible because there are huge differences”*
Belief in God	Strengthening in faith.	*“Don’t let yourself down, trust in God and your doctor!”*
Self-reflection	Reconsider your life.	*-*
Discreteness	Keep your illness as a secret.	*-*
Other	Statements not fitting in one of the other categories	*“Don’t forget being humble and grateful!”*

* categories which have been created based on the word by word analysis and the inductive analysis.

**Figure 1:**
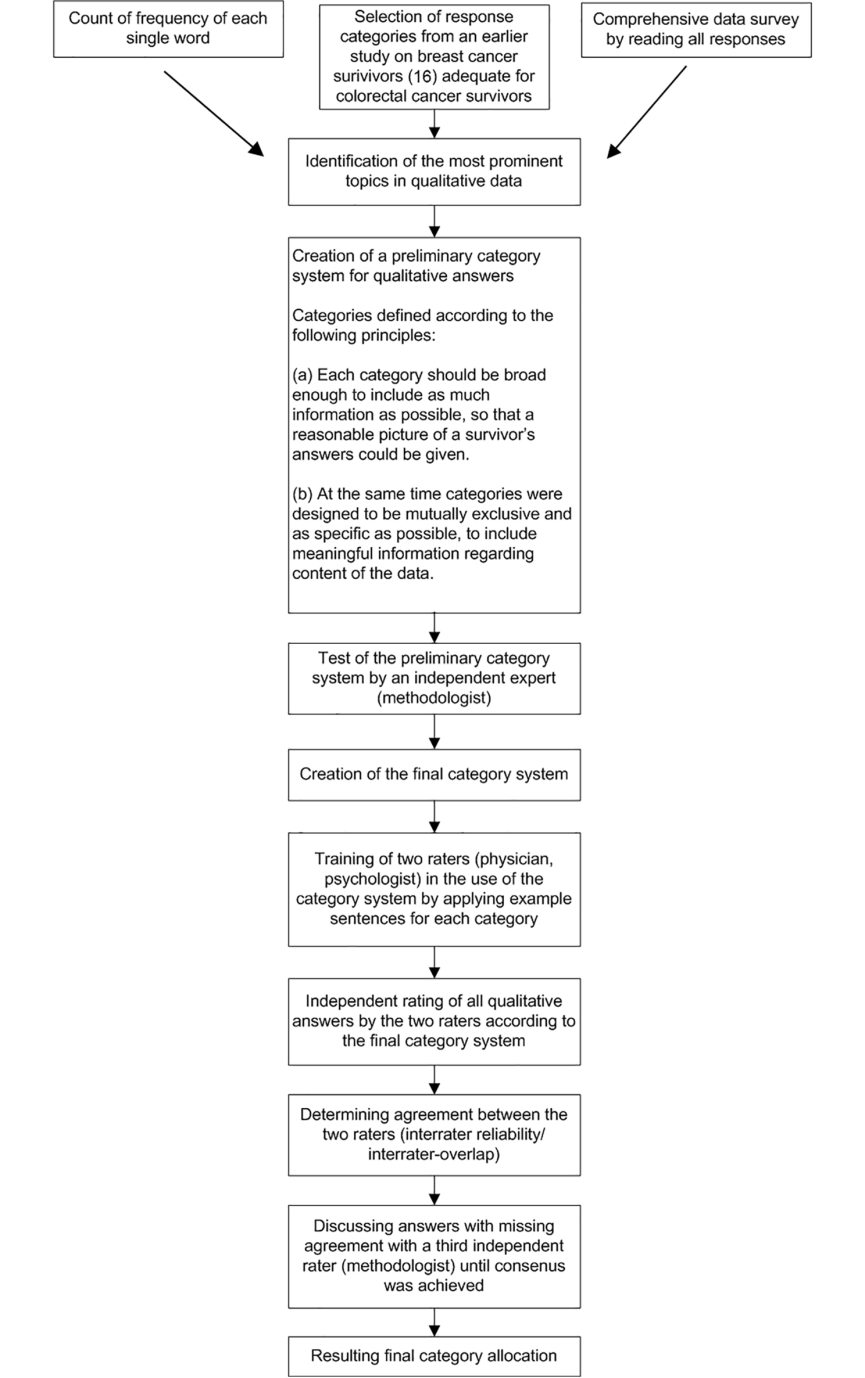
Sequence of each step in the qualitative data analysis

Two study authors (PLS and BS) were trained in the category system and rated all statements independently. In some cases, a participant’s answer was classified into more than one category. Finally, both investigators met with the expert methodologist (MK) to discuss divergent ratings until consensus was achieved. The whole process is visualised in Fig. 1.

### Statistical analysis

Agreement between the two raters was analysed using intercoder percent agreement and Cohen’s kappa to account for random agreement. Response categories were analysed quantitatively; descriptive statistics included frequencies, proportions, means, standard deviations (SD), medians, ranges, and interquartile ranges (IQR). In addition, the categories’ distribution was graphically compared to the distribution observed in the earlier study on breast cancer survivors from the same catchment area [20].

All data were analysed using the software package SPSS version 28 (IBM Corp., SPSS for Windows, Armonk, NY, USA). Some of the graphs were generated using R version 4.1.2 (R Foundation for Statistical Computing, Vienna, Austria) and the R package “plotrix”, version 3.8-2 [22].

## Results

### Participant characteristics

**Figure 2:**
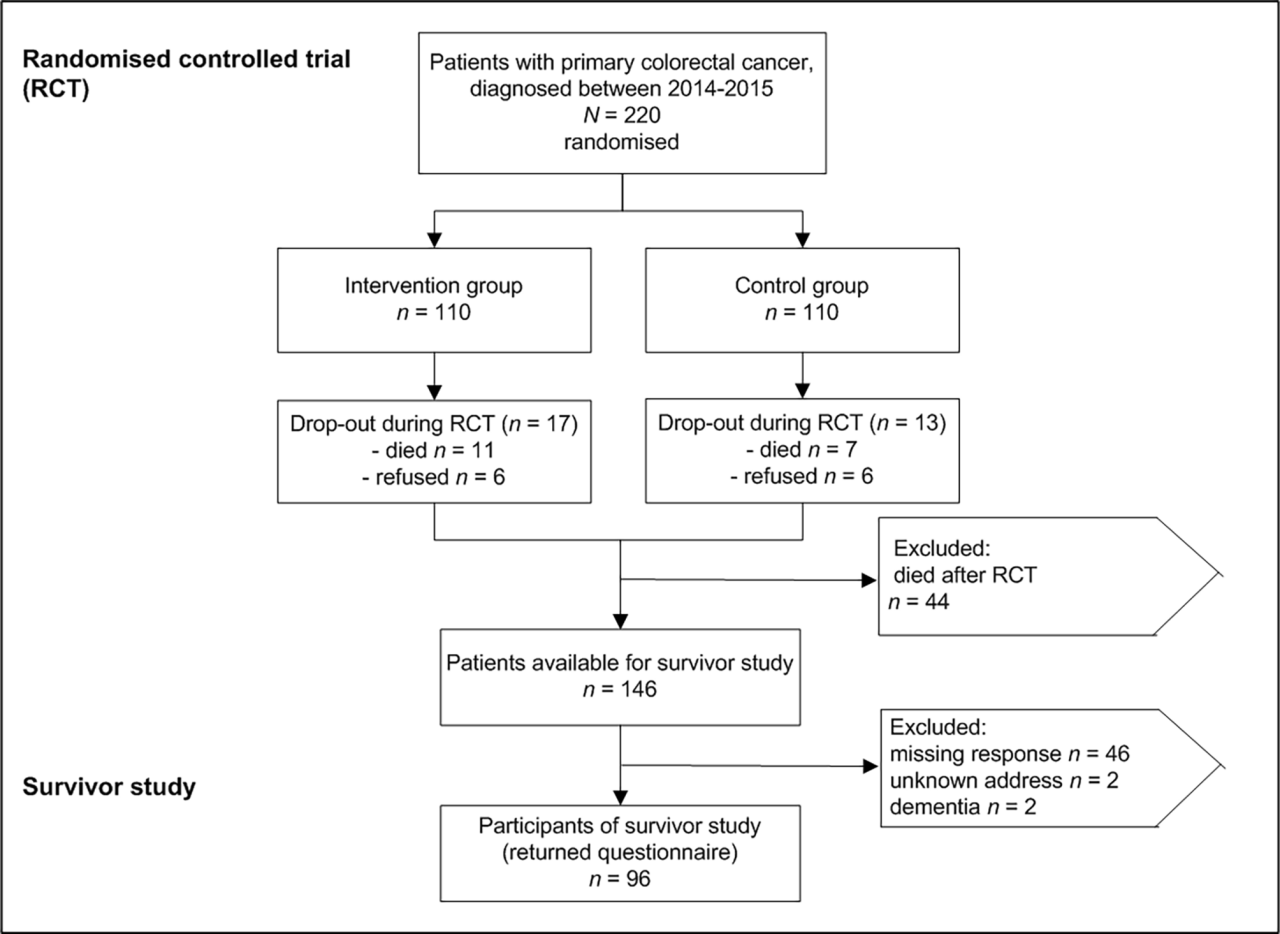
Patient allocation of the original RCT (DIQOL, (15)) and availability for the survivor study

Of 146 available survivors initially enrolled in the RCT DIQOL, 96 returned the questionnaire including both the quantitative QoL questionnaire (not reported in this paper) and the qualitative questions, corresponding to a response rate of 66% (cf. Figure 2). The mean age of participants at follow-up was 69·9 years (SD ± 10·3, range = 43–85) and the average interval between surgery and answering the follow-up questionnaire was 78·3 months (SD ± 5·5, range = 66–87). Further demographic and medical characteristics of participants are reported in Table 2.

**Table 2:** Demographic and medical characteristics of the participating colorectal cancer survivors (n = 96)

Time since surgery, mean (SD), months	78·3 (5·5)
Age at follow-up, mean (SD), years	69·9 (10·3)
	**No. (%) of participants**
Former intervention group patient (%)	45 (47)
Male (%)	65 (68)
Marital status at diagnosis	
Married	80 (83)
Unmarried	7 (7)
Widowed	5 (5)
Divorced	4 (4)
Children at diagnosis	
Yes	79 (82)
No	13 (14)
Unknown	4 (4)
Daily living (%)	
Urban area	33 (34)
Rural area	63 (66)
Prognostic stage at diagnosis	
UICC I	32 (33)
UICC II	17 (18)
UICC III	40 (42)
UICC IV	7 (7)
Primary site of disease	
Colon	51 (53)
Rectum	45 (47)
Surgical access	
Open	58 (60)
Laparoscopic	38 (40)
ASA at diagnosis^b^	
ASA I	17 (18)
ASA II	48 (50)
ASA III	29 (30)
ASA IV	-
Unknown	2 (2)
Comorbidities at diagnosis	
Cardiovascular	25 (26)
Kidney	8 (8)
Lung	12 (13)
Central nervous system	8 (8)
Stoma (%)	37 (39)
Reversed stoma	26 (70)
Preoperative symptoms	
Abdominal pain	26 (27)
Anaemia	18 (18)
Ileus	1 (1)
Bleeding	44 (46)
Neoadjuvant therapy	
Chemotherapy + radiotherapy	27 (28)
No neoadjuvant therapy	69 (72)
Adjuvant therapy (18 months after surgery)	
Chemotherapy	43 (45)
Antibody therapy	1 (1)
No adjuvant therapy	52 (54)

^a^ urban: Regensburg city and county, rural: Neumarkt, Straubing, Straubing-Bogen, Kelheim, Schwandorf.

^b^ American Society of Anesthesiologists.

### Analysis of response length and word frequency

Word length of participants’ responses was analysed: Longest answers were given when asked for *positive aspects* of their disease with a median of 7.5 words per answer (range 1–21 words, IQR: 5,75–10,25), while responses describing the *worst experience* were shortest with a median of 4.0 words per answer (range 1–20 words, IQR: 3–7). *Advice for future patients* was in between (word length median 6.0, range 1–26 words, IQR: 3–10).

To identify the most common issues in participants’ answers, frequencies of each single word were counted electronically. The four most frequent nouns addressing the *worst experience* were “surgery/ surgical” (n = 13), “anxiety” (n = 11), “stoma” (n = 10), and “diagnosis” (n = 10). Asked for *positive aspects*, survivors most frequently used the words “live/ living” (n = 7), “doctor/ medical” (n = 5), and “hospital” (n = 5). When giving *advice for future patients*, the most frequent words were “positive” (n = 17), “doctor/ medical” (n = 11), and “cancer screening” (n = 10).

### Categorisation

#### Worst experience

Eighty-one of 96 (84%) participants gave an answer to the question of their *worst experience*. The majority of statements was referring to “psychological distress”. Twenty-seven survivors of 81 (33%) gave a statement belonging to this category. Typical answers rated into this category were “fear of recurrence” or “the feeling of uncertainty”. With 14 associated statements (17%), “indigestion and discomfort during defecation” was the second most frequent category, while “cancer diagnosis” achieved the 3rd rank with n = 13 statements belonging to this category (16%).

However, considering only survivors who had received a stoma and answered the question, 10 out of 28 (36%) regarded “stoma” as their *worst experience*, rendering it the most frequently mentioned category in this group. Among survivors with a transient stoma, the share is even higher: Nine out of 23 (39%) of them regarded “stoma” as their *worst experience*.

**Figure 3:**
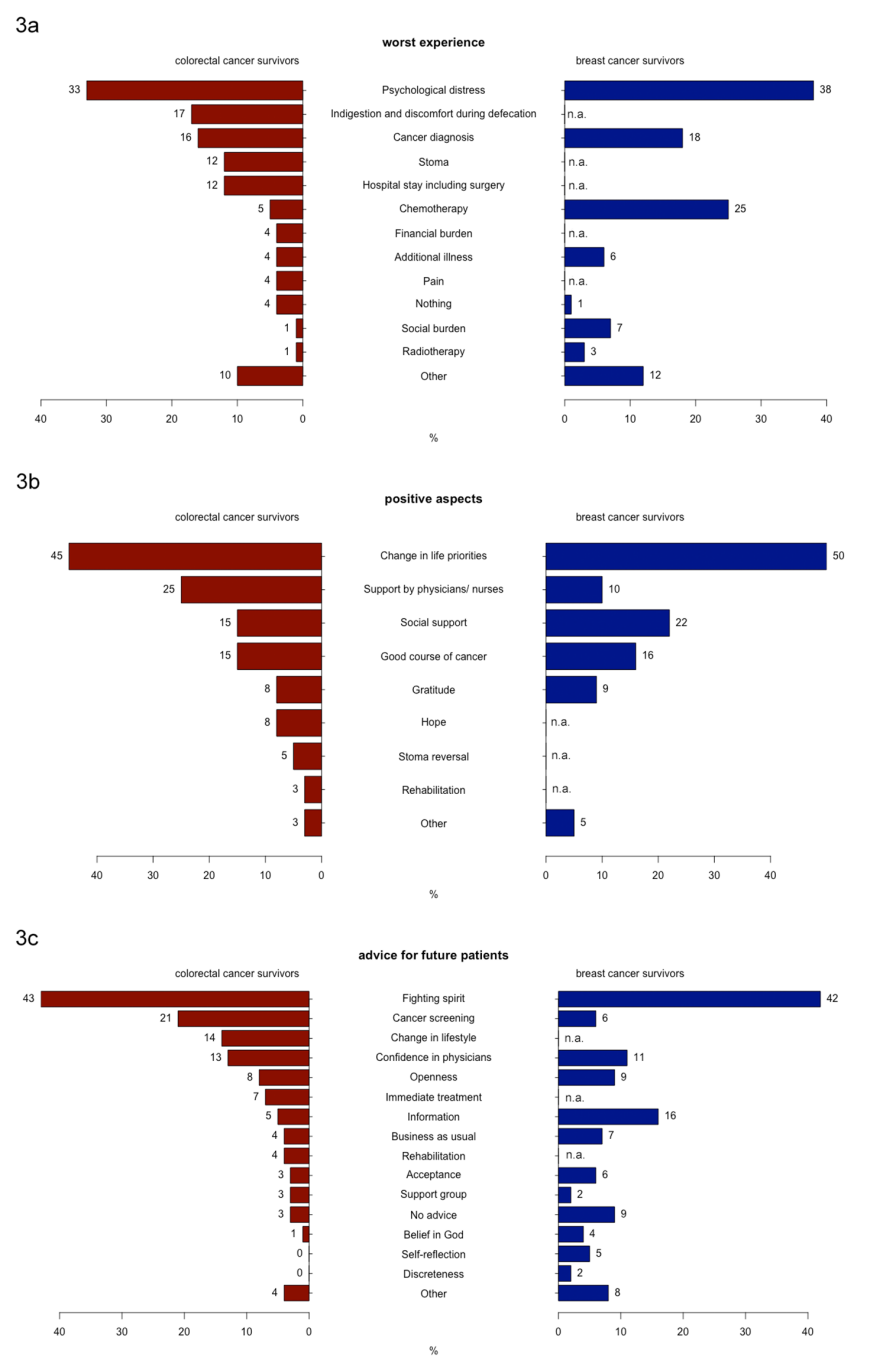
Percentaged distribution of the categorised statements of the colorectal cancer survivors’ (red) and breast cancer survivors’ (blue, [20]) concerning. (^a^ their worst experience,^b^ positive aspects of the illness, ^c^ advice for future patients)

Figure 3a gives a comprehensive overview of all categories contained in the answers for the *worst experience*; moreover, it contrasts the observed category-distribution to the results of the earlier study involving breast cancer survivors from the same catchment area. For the majority (38%) of them, “psychological distress” also represented the *worst experience* [20].

#### Positive aspects

Forty of 96 (42%) participants remembered *positive aspects* associated with their disease. Almost half of the colorectal cancer survivors who gave an answer to the corresponding question (n = 18, 45%) reported a “change in life priorities”, rendering it the most frequently mentioned category. Typical statements belonging to this category read like this: “I live more intensely. I have a more positive attitude towards many things”, “I care more for my body”, or “more healthy nutrition”. Ten out of 40 colorectal cancer survivors are grateful for the “support by physicians/ nurses” they obtained (25%). Some statements directly refer to a specific person like their surgeon. Figure 3b shows the frequencies of all mentioned categories; it also contains the corresponding results concerning *positive aspects* from the earlier breast cancer study, in which “change in life priorities” had been mentioned by 50% of the survivors, making it the most frequently mentioned *positive aspect*, too [20].

#### Advice for future patients

Seventy-seven of 96 (80%) participants had *advice for future patients.* The majority of them (n = 33, 43%) regards “fighting spirit” as most important aspect to overcome the disease. Concrete suggestions read like “think positive”, “don’t lose hope”, “believe in your odds”. Twenty percent advised to take part in “cancer screening”, making it the second most frequent suggestion. Figure 3c gives an overview of all mentioned categories together with the corresponding results from the earlier breast cancer study. Among the breast cancer survivors, “fighting spirit” had also been the most common *advice for future patients*, with 42% of the statements belonging to that category [20].

### Interrater percent agreement

There was an inter-rater percent agreement of 96% for category-allocation regarding *worst experience*, of 93% for category-allocation regarding *positive aspects*, and of 96% for category-allocation regarding *advice for future patients*. To quantify inter-rater-reliability, Cohen’s kappa was determined. The median kappa regarding *worst experience* was 0.79 (range 0.15–1.00), the median kappa regarding *positive aspects* was 0.73 (range 0.09–1.00), and the median kappa regarding *advice for future patients* was 0.68 (range − 0.13–1.00).

## Discussion

Even many years after successful tumour treatment, a lot of patients who survived colorectal cancer clearly remember certain aspects from the time of their illness and treatment. According to the present study, the majority of colorectal cancer survivors regards “psychological distress” as *worst experience*. Asked for *positive aspects* of the disease, the majority remembered “change in life priorities”; the most frequently given *advice for future patients* referred to “fighting spirit”. These categories had also been mentioned most frequently by the participants of an earlier study involving breast cancer survivors of the same catchment area [20].

### Results in the context of existing research

#### Worst experience

Of all three questions posted to the survivors, the answer-rate was highest concerning the *worst experience*. Since people regard cancer as most fatal diagnosis one can possibly get, it is not surprising that more survivors remember negative than positive aspects of their disease.

The majority of the survivors in this study regards “psychological distress” as *worst experience* in the course of their disease. Most statements rated into this category refer to anxiety. This supports the results of a large meta-analysis of Mitchell et al. [23] who showed that not general depression but uncertainty and fear are the biggest issues for cancer survivors and their spouses. It has been shown that fear of recurrence is associated with reduced emotional and social QoL [24]. This underlines the need to address this topic in patient-physician communication in order to identify patients requiring additional psychological support – especially in times, when unforeseen events like the COVID pandemic may lead to delayed follow-up examinations [25].

The fact that among survivors with history of a stoma this stoma is regarded as *worst experience* by survivors requires special attention: For many patients, a transient or permanent stoma is the only possibility to achieve a complete oncologic tumour resection. Therefore, additional efforts are necessary to support these patients in accepting this considerable change in their body image and in coping with accompanying discomforts like diarrhoea because otherwise they inevitably convert into “unhappy survivors”.

#### Positive aspects

Compared to the worst experience, the share of survivors reporting a positive experience is substantially lower. This stands in contrast to other studies, like the one of Sears et al. [26] which reported considerably higher rates (83%) of patients remembering positive experiences. Partly, this might be a consequence of the fact that Sears et al. exclusively included early-stage patients with excellent prognosis in their study, whereas the present study included patients of all stages. Another explanation for the observed difference might be that Sears et al. analysed recently diagnosed patients while the present study examined the attitude of long-term survivors. Looking at the most frequently mentioned *positive aspects*, colorectal cancer survivors regard change in life priorities as best experience in the course of their disease. This concept of ‘posttraumatic growth’ - personally important changes as a result of a life-threatening crisis – has often been described in relation to cancer survivorship [27] and can be regarded as coping strategy (giving sense to the disease), which is especially important at the time of diagnosis and during treatment. However, in the long run posttraumatic growth seems to play a less important role [28] and it can be also maladaptive if expectations of benefits are not realised [29].

#### Advice for future patients

The most frequently given advice of colorectal cancer survivors is to keep up a fighting spirit. In the present study the category fighting spirit included expressions in the sense of “think positive, fight, never lose hope, or be patient”. Studies with breast cancer patients showed that this kind of fighting spirit is associated with better psychological adjustment to the disease [30, 31]; other studies identified a positive relationship between trait optimism and well-being during treatment [32, 33] and long-term follow-up [34]. The popularity of the term “fighting spirit” may also be a consequence of the famous “war on cancer” [35], a topic often covered in the news media. In this context it is important to emphasize: While obviously many survivors regarded fighting spirit as an important prerequisite to overcome their cancer this does not mean that patients who “lost” their personal “war” did not fight hard enough or are even to blame. Understanding the advice “fighting spirit” that way would be more than counterproductive.

### Comparison to an earlier survey on breast cancer survivors

In 2015, Lindberg et al. already published a study on long-term recollections of breast cancer survivors of the same catchment area, which had been designed according to the same methodology as the present study. The share of breast cancer survivors willing to share their *worst experience*, to give insight in *positive aspects*, and to give *advice to future patients* had been 94%, 48%, and 88%, respectively [20], which is quite comparable to the shares observed in the present study.

#### Worst experience

In the previous breast cancer survivors’ survey [20], 38% of the participants were referring to “psychological distress” as *worst experience*, which is a similar share like in the present study. However, 25% of the breast cancer survivors regarded “chemotherapy” as their second *worst experience*, which is most probably a consequence of the perception of the medical side effects [36]. For colorectal cancer survivors, chemotherapy was of a considerably lower importance, although chemotherapy rates over all stages are comparable [37, 38] and side effects are also a common issue. A reason for this might be that breast cancer patients are usually younger, female, and not accustomed to physical impairments. Symptoms like fatigue, oedema, or chemotherapy-associated alopecia suddenly restrict their physical capability or lead to a disturbed body-image with consequences e.g. for their professional or social life, while the usually older and often already retired colorectal cancer patients might already have learned to deal with comparable symptoms due to existing non-oncologic comorbidities. Another explanation could be that the side effects of chemotherapy seem less severe if a patient is confronted with permanent indigestion or surgical complications like wound infection or anastomotic insufficiency at the same time.

#### Positive aspects

Concerning *positive aspects*, “change in life priorities” had also been the most frequent category in the breast cancer survivors’ survey with a 46% share [20], while statements belonging to the category “support by physicians/ nurses”, the second most frequently mentioned category of the present study, were only given by a lower share of 10% among breast cancer survivors [20]. For breast cancer survivors the “social support” they received was more important with a share of 22% vs. 6% among the colorectal cancer survivors (cf. Figure 3b). This might again be a consequence of the higher mean age of the colorectal cancer survivors group. The social network of older patients tends to be smaller and often consists of individuals who suffer from health problems themselves and have less capacities to support others. Thus, the older colorectal cancer patients almost automatically focus more on their physicians or nurses. This underlines the importance of establishing a good patient-caregiver relationship [39].

#### Advice for future patients

The majority of breast cancer survivors deemed “fighting spirit” as most important *advice for future patients* [20]; Fourty-two percent gave a statement belonging to this category, which is almost the same share like observed among the colorectal cancer survivors [20]. In contrast to the results seen in this study, the second most frequent advice of breast cancer survivors (16%) was to look for “information”, which has only been suggested by 5% of the colorectal cancer survivors (cf. Figure 3c). On the other hand, with a share of 6%, only a few breast cancer survivors deemed “cancer screening” an important *advice for future patients* [20]. The fact that more colorectal than breast cancer survivors advocated for participation in screening programmes to detect the tumour in an earlier, easier to treat stage is definitely remarkable. In Germany, every woman between 50 and 69 gets automatically invited to regular mammographies, whereas the decision for a colonoscopy requires more self-initiative. Nevertheless, it seems like colorectal cancer patients are more convinced of the efficacy of “their” screening programme. This topic requires future research.

### Strengths and limitations

The study population was clearly defined due to the survivors’ participation in an earlier RCT [15], which was representative for patients stemming from both urban and rural areas.

Being aware of potential distortions like recall and hindsight bias [40], reframing [41], and response shift [42, 43], we deliberately bypassed the issue of whether survivors accurately remembered experiences that cannot be objectively confirmed. We rather decided to focus on the survivors’ subjective recollections. By communicating their opinions and experiences to others, survivors contribute to create the perceived stereotypes of cancer, which, in turn, will influence future generations of patients [44].

We applied a data-driven approach to categorize patients’ responses to open-ended questions, used a pair of independent raters that have been trained in the category system and had a third methodologist available to reach consensus and establish a high degree of interrater congruence. This objective, data-driven method to analyse verbal data avoids the subjectivity of a purely “qualitative analysis”. Nevertheless, each category will contain a range of similar but of course not entirely identical perspectives. However, the high congruence with regards to content between the frequencies of the different categories and the frequencies of the most common words within the patients’ answers is an indicator of a high degree of objectivity.

There are also some limitations: As a consequence of using a mailed survey instead of face-to-face interviews, the qualitative answers were sometimes short and enquiry for further explanation was not possible. On the other hand, a mailed survey has the advantage of a lower likelihood of social desirability than in interviews, the avoidance of an interviewer’s possible influence, and the accessibility of a larger sample of participants. Furthermore, it has to be acknowledged that out of the original 220 patients enrolled in the RCT, only n = 96 were available for this analyses. This certainly had an impact on relative distribution of answer categories. Thus, our results represent the perspectives of colorectal cancer patients who have still been alive at the time of this follow-up study and were also willing to participate.

## Conclusion

Understanding long-term recollections of cancer survivors is very important to minimise the negative impact of a tumour disease on a patient. While the most frequently mentioned worst long-term recollection “psychological distress” and the most frequently given advice “fighting spirit” are often mentioned concepts in the general context of cancer, there are other aspects like the impact of a stoma with specific importance for colorectal cancer survivors. This information can help to improve communication with cancer patients and inform the future implementation of corresponding cancer-specific therapeutic and supportive programmes to monitor, preserve, and improve QoL during therapy and (long-term) aftercare of tumour diseases.

## Data Availability

Data containing potentially identifying or sensitive patient information are restricted according to European law (General Data Protection Regulation (GDPR)). The datasets generated and analysed during the current study are not available in a public repository due to its inclusion of health information but are available upon reasonable request from Monika Klinkhammer-Schalke.

## Abbreviations

DIQOL: Direct improvement of quality of life
IQR: interquartile range
PRO: Patient reported outcome
QoL: Quality of life
RCT: Randomised controlled trial
SD: standard deviation
